# Evaluation of the eclipse electron Monte Carlo dose calculation for small fields

**DOI:** 10.1120/jacmp.v10i3.2834

**Published:** 2009-07-09

**Authors:** Zhigang Xu, Sarah E. Walsh, Tejas P. Telivala, Allen G. Meek, Guozhen Yang

**Affiliations:** ^1^ Department of Radiation Oncology Stony Brook University Medical Center Stony Brook New York; ^2^ Department of Medical Physics Memorial Sloan‐Kettering Cancer Center Commack New York

**Keywords:** electron beam, Monte Carlo, small fields, MU calculation, extended SSD

## Abstract

Varian Medical Systems (Palo Alto, CA) has implemented the Monte Carlo electron dose calculation algorithm (eMC) in the Eclipse treatment planning system. Previous algorithms for electron treatment planning were limited in their calculation ability for small field depth doses and monitor units. An old rule of thumb to approximate the limiting cutout size for an electron field was determined by the lateral scatter equilibrium and approximated by E (MeV)/2.5 in centimeters of water. In this study, we compared eMC calculations and measurements of depth doses, isodose distributions, and monitor units for several different energy and small field cutout size combinations at different SSDs. Measurements were made using EBT film (International Specialty Products, Wayne, NJ) and a PinPoint ion chamber (PTW‐New York Corp., Hicksville, NY). Our results indicate that the eMC algorithm can accurately predict depth doses, isodose distributions, and monitor units (within 2.5%) for field sizes as small as 3.0 cm diameter for energies in the 6 to 20 MeV range at 100 cm SSD. Therefore, the previous energy dependent rule of thumb does not apply to the Eclipse electron Monte Carlo code. However, at extended SSDs (105–110 cm), the results show good agreement (within 4%) only for higher energies (12, 16, and 20 MeV) for a field size of 3 cm.

PACS number: 87.53.Hv

## I. INTRODUCTION

Most commercial treatment planning systems incorporate electron beam planning programs. However, not all programs have comparable accuracy or limitations. Electron beam dose calculations were originally based on empirical functions that utilized ray line geometries and assumed broad beam dose distributions in homogeneous media.^(1)^ More advanced Pencil Beam algorithms, based on multiple scattering theories, were developed in the early 1980s by Hogstrom et al.^(2)^ One major limitation of both the empirical methods and the Pencil Beam algorithms is their inability to predict depth dose distributions and accurate monitor units for field sizes smaller than the extent of lateral scatter equilibrium. The approximation for lateral scatter equilibrium was determined by Lax and Brahme to be E (MeV)/2.5 in centimeters of water.^(3)^ A rule of thumb it that treatment planning systems cannot accurately predict clinically relevant dosimetric data for cutout diameters less than the Lax and Brahme approximation.

A commercial Monte Carlo‐based dose calculation algorithm has become available for electron beam treatment planning in the Varian Eclipse treatment planning system. The accuracy of implementation of this algorithm in Eclipse was investigated by several groups.^(^ ^4^ ^–^ ^6^ ^)^ We report here on the evaluation of this commercial product by comparison of calculations with measurements performed at our institution for small field sizes. With the implementation of this new calculation algorithm, we investigated if the old rule of thumb could be upheld or if a new rule of thumb could be determined.

## II. MATERIALS AND METHODS

### A. Calculations

Varian Eclipse electron Monte Carlo (eMC) algorithm is a fast implementation of the Monte Carlo method for dose distribution calculation from high energy electron beams in radiotherapy treatment planning. The algorithm consists of:

- Electron transport/dose deposition model (transport model, Macro Monte Carlo method^(5)^) performing the transport and dose deposition caused by the electrons in the patient
- Electron beam phase‐space model (Initial Phase Space model, IPS) describing the electrons that emerge from the treatment head of the linear accelerator

The eMC has six user‐selectable parameters for individual calculations: calculation grid size, accuracy, maximum number of particle histories, random number generator seed, smoothing method, and smoothing level.^(6)^ To attain accurate calculations and consistency within a reasonable amount of time, the eMC calculation parameters used in this study are listed in Table 1. Based on research done by Popple et al.^(6)^ to achieve clinically acceptable results, grid sizes for eMC calculations should be varied based on energy. The grid size should be approximately one‐tenth of the distal falloff distance of the electron depth dose curve (depth from 80% to 20% of the maximum dose). A typical eMC calculation takes about 2 minutes on a 2.6 GHz CPU for a 5 cm circular cutout (12 MeV) at 100 cm SSD with 1% accuracy and 1.5 mm grid size.

**Table 1:** Eclipse electron Monte Carlo calculation parameters used in this study.

*Parameter*	*Values*
Calculation grid size	1 mm (6 MeV, 9 MeV)
	1.5 mm (12 MeV)
	2 mm (16 MeV)
	2.5 mm (20 MeV)
Accuracy	1%
Maximum number of particle histories	0 (calculates until desired accuracy goal is reached
Random generator seed number	1 to 3100000000
Smoothing method	3D Gaussian
Smoothing level	1‐Low

To evaluate the Varian Eclipse electron Monte Carlo (eMC) algorithm performance for small field sizes, calculations of depth doses, isodose distributions, and monitor units were done for all energies available at our institution (6, 9, 12, 16 and 20 MeV) in a water equivalent phantom created in Eclipse. Dose distributions and monitor units were calculated for standard $10\times 10\;{\text{cm}}^{2}$ open field as well as five cerrobend circular cutout sizes (5, 4, 3, 2 and 1 cm diameters) using $10\times 10\;{\text{cm}}^{2}$ and $6\times 6\;{\text{cm}}^{2}$ cones at 100 cm Source to Surface Distance (SSD). Calculations were also performed for cutout size of 2 and 3 cm at extended SSDs for all energies. For fields that are long and narrow, such as 2cm×9cm and 3cm×9cm rectangles, calculations were done at 100 cm SSD for all energies.

Initial eMC plans were created in Eclipse for each cutout size and energy combination without normalization or prescription points. Dose maximum values were determined in these plans by using Eclipse's vertical dose profile tool along the central axes. We prescribed 100 cGy to each dose maximum depth, $d_{\text{max}}$, and calculated monitor unit values. The plans were normalized to 100% at their respective $d_{\text{max}}$ for analysis.

### B. Measurements

**Figure 1:**
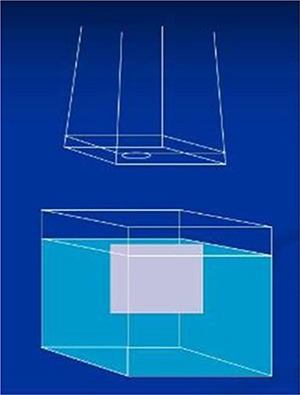
Water tank setup used for film measurements. Gafchromic EBT film was lined up to the surface of the water set to different SSDs and held in place by adjustable clamps (not shown). Film was aligned to bisect the electron cutout placed in the electron applicator.

In this experiment, we used the latest product development in Gafchromic film called EBT (International Specialty Products, Wayne, NJ) to obtain central axis depth doses as well as planar dose distributions for all energy and cutout combinations. Earlier versions of Gafchromic film were used for many years in radiotherapy as a dosimeter and QA device.^(^ ^7^ ^,^ ^8^ ^)^ Limitations in earlier Gafchromic products, such as energy dependence and sensitivity range, led to the development of this new film. In 2004 Gafchromic EBT film was launched as a clinically energy independent film with a sensitivity range of 1 to 800 cGy.^(9)^ It is a more versatile dosimeter and QA device than its predecessors.^(^ ^10^ ^,^ ^11^ ^)^ One characteristic of EBT film is its ability to be submerged in water for up to an hour without harming its integrity.^(12)^ This characteristic was essential for our setup (Fig.1). We submerged pieces of film in a water tank and aligned their top edge to the surface of the water. The film was positioned to bisect the electron cutout and held in place by adjustable clamps (not shown in Fig.1). With this setup we were able to perform accurate vertical dosimetry.

All measurements were performed on a Varian Silhouette Edition Clinac SiL21IX (Varian Medical Systems, Palo Alto, CA) linear accelerator. An optical density versus dose calibration curve was obtained for each film batch used in the study. The film was calibrated using 6 MV photons. Although the energy range for the electron beams in this study is 6MeV to 20 MeV. calibration of the film using 6 MV photons was valid since the energy dependence was minimal^(9)^ Films were placed at the depth of dose maximum (1.5 cm) in a solid water phantom and irradiated with doses ranging from 10–300 cGy. While absolute dosimetry is possible with this film, we used it as a relative dosimeter.

The accuracy of monitor unit (MU) calculation is an important feature of any treatment planning system. We verified the accuracy of dose monitor unit calculations by comparing calculated and measured MUs needed for delivering 100 cGy at a depth of maximum dose. $d_{\text{max}}$, in a water phantom. Based on the measurements, MUs needed for delivering 100 cGy at $d_{\text{max}}$ were calculated according to:

(1) $$MU=\frac{100\times D_{ref}(10\times 10\;{cm}^{2},10\times 10\;cone,SSD=100\;cm)}{D_{\text{cutout}}\;(c,A,SSD)}$$

where $D_{\text{cutout}}$ (c, A, SSD) is the dose at $d_{\text{max}}$ on the central axis for a cutout size *c*, in an applicator *A*, and a source‐to‐surface distance SSD, per 100 MU delivered, and $D_{\text{ref}}(10\times 10\;{\text{cm}}^{2},\;10\times 10\;{\text{cm}}^{2}\;\text{cone}$, SSD=100cm) is the reference dose at $d_{\text{max}}$ for an open $10\times 10\;{\text{cm}}^{2}$ applicator on the central axis for 100 MU delivered.^(4)^

Measurements were done using a PinPoint ion chamber‐ T31006 (0.015 cm^3^ active volume, 2 mm active diameter, 5 mm active length) (PTW, Hicksville, NY) in a water tank. The PinPoint ion chamber was positioned at the $d_{\text{max}}$ values determined by the Eclipse treatment planning system for each cutout size and energy combination at different SSDs.

### C. Film Analysis

Depth dose comparisons of the Eclipse dose planes and EBT films were done using the film analysis software FilmQA(3Cognition, Great Neck, NY) and Excel (Microsoft, Redmond, WA). Vertical dose planes from Eclipse calculations were imported into FilmQA. The EBT films were scanned into the software with an Epson 10000XL flatbed color scanner, in a method similar to that described by Wilcox et al.^(9)^ This software was designed to correctly analyze EBT film by extracting its red color channel information from the RGB scan of the film. The peak absorption of EBT film is in the red region of the visible spectrum.^(10)^ A background correction was done to correct for non‐uniformities of the scanner. An unexposed piece of film was scanned and images were corrected on a pixel by pixel basis by FilmQA. After importing into FilmQA, corresponding images were registered. In the “Evaluate” section of FilmQA depth dose curves along the central axis were extracted from the planar dose distributions and normalized to 100% at $d_{\text{max}}$. The data for these curves was exported into Excel and graphed so we could compare the relative match of the Eclipse calculations and the EBT films. For each cutout, we created one graph which included all calculations and measurements for every energy level tested. Quantitative analysis was done by measuring the distances between the depth dose curves at every 5% intervals between 20% and 80% dose.

To provide comparisons of multidimensional dose distributions, dose comparison tools such as gamma dose distribution, distance‐to‐agreement (DTA), and dose difference (DD) have been developed^(^ ^13^ ^,^ ^14^ ^)^ FILMQA includes a selection of evaluation tools that can be used to compare the dose distribution of the films to the dose distribution of the calculations. The gamma map is a qualitative map that is a mathematical combination of the dose difference and distance‐to‐agreement calculations. The gamma dose distribution tool was used in our film analysis. We compared sagittal isodose distributions along the central axis and performed a gamma analysis for each energy/cutout combination in FilmQA. Our acceptable gamma pixel parameters were set to 5% dose difference and 3 mm distance‐to‐agreement. The choice of 5% and 3 mm as comparison criteria was somewhat arbitrary and was used in our routine clinical practice.

## III. RESULTS & DISCUSSION

**Figure 2:**
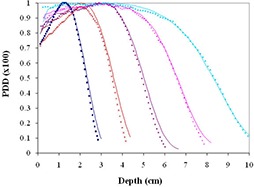
Percent depth dose curve comparisons between eMC calculations and EBT in water measurements for $10\times 10\;{\text{cm}}^{2}$ open field at 100 cm SSD using $10\times 10\;{\text{cm}}^{2}$ cone. All curves are normalized to 100%. The Eclipse measurements are represented by solid lines while the EBT measurements are represented by dotted lines as follows: 6 MeV (dark blue), 9 MeV (brown), 12 MeV (purple), 16 MeV (magenta), 20 MeV (light blue).

Figure 2 shows the comparison between the calculated and measured percentage depth dose curves for beams of energy 6, 9, 12, 16 and 20 MeV for a $10\times 10\;{\text{cm}}^{2}$ open field in water at 100 cm SSD using $10\times 10\;{\text{cm}}^{2}$ cone. The eMC calculations are shown as solid lines and the EBT film measurements are shown as dotted lines. The mean distance discrepancies (Δ in mm) between calculation and measurement doses evaluated at 5% intervals between 20% and 80% dose are given in Table 2. The corresponding standard deviation, σ, is given in parentheses. The agreement between calculated and measured data was excellent (within 1 mm), thereby validating our setup.

**Table 2:** Mean distance discrepancies (Δ in mm) between calculation and measurement doses evaluated at 5% intervals between 20% and 80% dose range for beams of energy 6, 9, 12, 16 and 20 MeV for a $10\times 10\;{\text{cm}}^{2}$ open field and cutout sizes of 5, 4, 3, 2 and 1 cm in water at 100 cm SSD using $10\times 10\;{\text{cm}}^{2}$ cone. The corresponding standard deviation, σ, is given in parentheses.

		*Electron Beam Energies*			
*Cutout size*	*6 MeV*	*El 9 MeV*	*12 MeV*	*16 MeV*	*20 MeV*
10×10cm	0.7 (0.3)	1.0 (0.4)	1.0 (0.4)	0.5 (0.3)	0.4 (0.2)
5 cm	0.5 (0.4)	0.2 (0.2)	0.5 (0.2)	0.3 (0.2)	1.3 (0.8)
4 cm	0.5 (0.4)	0.4 (0.2)	0.5 (0.3)	0.3 (0.2)	0.4 (0.3)
3 cm	0.4 (0.3)	0.3 (0.2)	0.5 (0.4)	0.6 (0.3)	1.2 (0.7)
2 cm	0.3 (0.1)	0.7 (0.3)	1.8 (1.0)	1.6 (1.2)	2.1 (1.3)
1 cm	2.9 (1.1)	5.6 (2.0)	8.3 (1.7)	11.4 (3.8)	16.8 (6.2)

**Figure 3:**
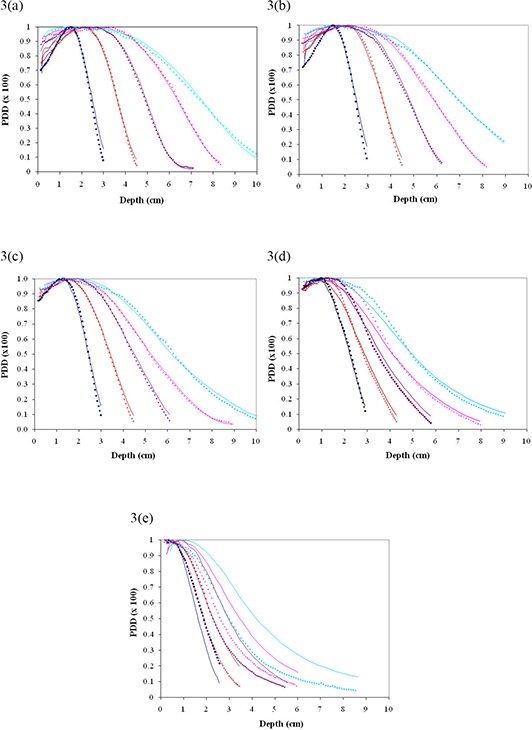
(a) – 3(e). Percent depth dose curve comparisons between eMC calculations and EBT in water measurements for 5, 4, 3, 2 and 1 cm cutout, respectively, at 100 cm SSD using $10\times 10\;{\text{cm}}^{2}$ cone. All curves are normalized to 100%. The Eclipse measurements are represented by solid lines while the EBT measurements are represented by dotted lines as follows: 6 MeV (dark blue), 9 MeV (brown), 12 MeV (purple), 16 MeV (magenta), 20 MeV (light blue).

The comparison between calculated and measured percentage depth doses for cutout sizes of 5, 4, 3, 2 and 1 cm are shown in Fig. 3(a) – 3(e) for beams of energy 6, 9, 12, 16 and 20 MeV in water at 100 cm SSD using $10\times 10\;{\text{cm}}^{2}$ cone. The mean distance discrepancies (Δ in mm) and corresponding standard deviations σ are listed in Table 2, as well. The calculations matched the EBT film results within 1.3 mm on average for 5, 4 and 3 cm cutouts over all energies tested as indicated in the Table 2. The 2 cm cutout matched fairly well with the largest mean distance discrepancy of 2.1 mm for 20 MeV. The 1 cm cutout, shown in Fig. 2(e), gave no discernable matches.

**Figure 4:**
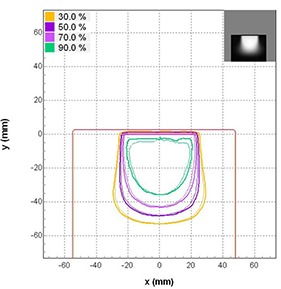
Sagittal isodose comparisons of a 12 MeV beam along the central axis for 5 cm cutout at 100 cm SSD using $10\times 10\;{\text{cm}}^{2}$ cone. Thick lines represent eMC calculations and thin lines represent EBT film measurements. The 30, 50, 70, and 90 percentages are shown.

**Figure 5:**
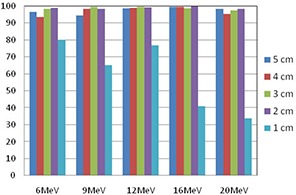
Gamma analysis results for beams of energy 6, 9, 12, 16 and 20 MeV for cutout sizes of 5, 4, 3, 2 and 1 cm in water at SSD=100cm using $10\times 10\;{\text{cm}}^{2}$ cone.

Our isodose distributions and gamma analyses showed findings similar to the depth doses comparisons. A typical isodose distribution for 12 MeV and cutout size of 5 cm at 100 cm SSD using $10\times 10\;{\text{cm}}^{2}$ cone is shown in Fig. 4. The 90%, 70%, 50% and 30% isodose lines are shown. The thick lines represent the calculated doses, while the thin lines represent the measured doses obtained from the EBT film. Similar results were obtained for other beams of energy and cutout sizes. All comparisons matched well except in the case of the 1 cm cutout. Gamma analysis results for all energies and cutout sizes are shown in Fig. 5. The agreement between measured and calculated values was excellent for the 5, 4, 3 and 2 cm cutouts, with greater than 93.4% of pixels passing our gamma requirements for all energies tested. The 1 cm cutout showed poor results; the numbers of pixels passing our gamma requirements were below 80% for all the energies tested.

Comparisons of calculated and measured monitor units for beams of energy 6, 9, 12, 16 and 20 MeV and cutout sizes of 5, 4, 3, 2 and 1 cm at 100 cm SSD using $10\times 10\;{\text{cm}}^{2}$ cone are listed in Table 3. The agreement of MUs was very good (within 2.4%) for cutout sizes of 5, 4, and 3 cm for all tested energies. For the 2 cm cutout, the calculated MUs agreed with measurements to within 5% for all tested energies. The 1 cm cutout presented the worst results with percent differences above 8%.

**Table 3:** Percent difference between calculated and measured monitor units for beams of energy 6, 9, 12, 16 and 20 MeV for cutout sizes of 5, 4, 3, 2 and 1 cm in water at 100 cm SSD using $10\times 10\;{\text{cm}}^{2}$ cone.

		*Electron Beam Energies*			
*Cutout size*	*6 MeV*	*9 MeV*	*12 MeV*	*16 MeV*	*20 MeV*
5 cm	1.2	0.5	0.3	0.0	1.4
4 cm	1.9	1.6	0.4	0.5	1.5
3 cm	1.2	0.6	1.2	1.2	2.4
2 cm	3.9	4.3	4.7	2.6	3.9
1 cm	43.5	34.3	24.9	13.5	8.2

**Figure 6:**
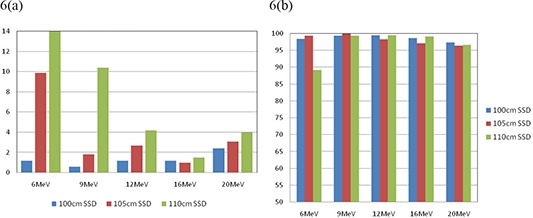
Comparison of calculated and measured (a) MU% difference, and (b) gamma analysis results for 3 cm cutout size at extended SSDs for all tested energies using $10\times 10\;{\text{cm}}^{2}$ cone.

We also tested the accuracy of monitor unit calculations and dose distribution calculations for commonly used extended SSDs (105–110 cm) for small cutout sizes for all available energies. Figures 6(a) and 6(b) show the comparison between the calculated and measured MUs and dose distributions for 3 cm cutout sizes at SSD=100,105 and 110 cm. At 105 cm SSD, the calculated MUs were all within 3.1% of measured values, except in the case of the 6 MeV beam. In this case, the MU% difference was 10% whereas the dose distributions agreed well with greater than 99.3% of pixels passing our gamma requirements. Significant differences (<10%MU difference, <90%pixel passing rate) exist between calculated and measured MUs and dose distributions at 110 cm SSD for energies of 6 and 9 MeV for cutout size of 3 cm. This is in agreement with the findings reported by Ding et al.^(4)^

**Figure 7:**
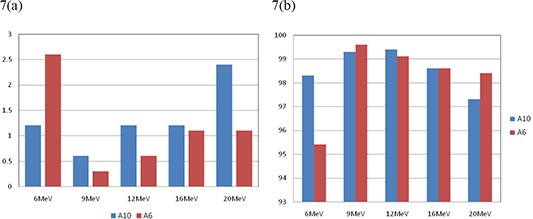
Comparison of calculated and measured (a) MU% difference, and (b) gamma analysis results for 3 cm cutout size at 100 cm SSD for all tested energies using $10\times 10\;{\text{cm}}^{2}$ and $6\times 6\;{\text{cm}}^{2}$ cones.

**Figure 8:**
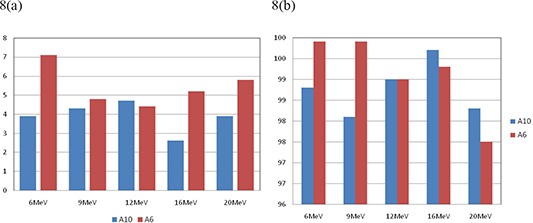
Comparison of calculated and measured (a) MU% difference, and (b) gamma analysis results for 2 cm cutout size at 100 cm SSD for all tested energies using $10\times 10\;{\text{cm}}^{2}$ and $6\times 6\;{\text{cm}}^{2}$ cones.

It is noteworthy that similar results were obtained using $6\times 6\;{\text{cm}}^{2}$ cone. Figures 7(a) and 7(b) show the comparison between $10\times 10\;{\text{cm}}^{2}$ and $6\times 6\;{\text{cm}}^{2}$ cones for 3 cm cutout size at SSD=100cm. The agreement of MUs was very good (within 2.5%) for all tested energies for both $10\times 10\;{\text{cm}}^{2}$ and $6\times 6\;{\text{cm}}^{2}$ cones. The corresponding dose distributions agreed well with greater than 95% of pixels passing our gamma requirements using either cone. However, for a 2 cm cutout, as shown in Fig. 8(a) and 8(b), the calculated MUs agreed with measurements on the average of 5% and with greater than 97% of pixels passing our gamma requirements for all tested energies using either $10\times 10\;{\text{cm}}^{2}$ or $6\times 6\;{\text{cm}}^{2}$ cone.

**Figure 9:**
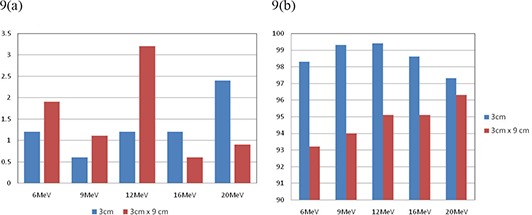
Comparison of calculated and measured (a) MU% difference, and (b) gamma analysis results for cutout sizes of 3 cm circle and 3cm×9cm at 100 cm SSD using $10\times 10\;{\text{cm}}^{2}$ cone for all tested energies.

**Figure 10:**
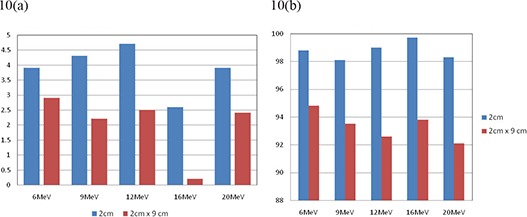
Comparison of calculated and measured (a) MU% difference, and (b) gamma analysis results for cutout sizes of 2 cm circle and 2cm×9cm at 100 cm SSD using $10\times 10\;{\text{cm}}^{2}$ cone for all tested energies.

In clinical practice, fields that are long and narrow are about as common as fields that are small in both dimensions. We also investigated such fields, namely 3cm×9cm and 2cm×9cm rectangles. The results are shown in Fig. 9(a) and 9(b) and Fig. 10(a) and 10(b), respectively. These results suggest that there are no significant differences between circular and rectangular shape in terms of comparison between calculated and measured results. As long as the shorter dimension of a shaped field is smaller than 3.0 cm, calculated dose distribution and MUs can be differed significantly from the measurement.

## IV. CONCLUSIONS

In conclusion, we compared eMC calculations and measurements of depth doses, isodose distributions, and monitor units for several different energies and small field cutout size combinations at different SSDs. Our results show that the Monte Carlo algorithm for electron planning in Eclipse is more accurate than previous algorithms for small field sizes in homogenous mediums. We believe the minimum cutout size eMC can accurately predict depth doses, isodose distributions, and monitor units for as small as a 3 cm diameter for energies in the 6 to 20 MeV range at 100 cm SSD, consistent with the recommendation of Popple et al.^(6)^ Therefore, the previous energy dependent rule of thumb does not apply to the Eclipse electron Monte Carlo code. When a cutout size or any dimension of a shaped field is smaller than 3.0 cm, calculated dose distribution and MUs can differ significantly from the measurement. At extended SSDs (105–110 cm), the results show good agreement (within 4%) only for higher energies (12, 16, and 20 MeV) for a field size of 3 cm. As Monte Carlo‐based treatment planning systems begin to enter clinical practice, one should pay particular attention to those fields with cutout sizes smaller than 3 cm in diameter or at extended SSDs with low energies. In such cases, a special dosimetry (e.g. output factor, depth‐dose, and isodose distribution) should be measured and used for the treatment planning.
